# New Delhi Metallo-β-Lactamase from Traveler Returning to Canada^1^

**DOI:** 10.3201/eid1702.101313

**Published:** 2011-02

**Authors:** Gisele Peirano, Jasmine Ahmed-Bentley, Neil Woodford, Johann D. Pitout

**Affiliations:** Author affiliations: Calgary Laboratory Services, Calgary, Alberta, Canada (G. Peirano, J.D. Pitout);; University of Calgary, Calgary (G. Peirano, J.D. Pitout);; DynaLife Diagnostic Laboratories Services, Edmonton, Alberta, Canada (J. Ahmed-Bentley);; Health Protection Agency, London, UK (N. Woodford)

**Keywords:** Urinary tract infection, New Delhi metallo-β-lactamase, Escherichia coli, bacteria, antimicrobial drug resistance, carbapenems, nosocomial, Canada, expedited, dispatch

## Abstract

An *Escherichia coli* isolate with New Delhi metallo-β-lactamase was isolated from a patient with pyelonephritis and prostatitis who returned to Canada after recent hospitalization in India. The patient was successfully treated with ertapenem and fosfomycin. This patient highlights the role of international travel in the spread of antimicrobial drug resistance and *bla*_NDM-1_.

The *Enterobacteriaceae*, particularly *Escherichia coli* and *Klebsiella pneumoniae*, are among the most common causes of serious hospital- and community-acquired bacterial infections in humans. Resistance to antimicrobial agents in these species has become increasingly prevalent. Of special concern is the development of resistance to the carbapenems; this development is caused by bacterial carbapenemases. These drugs are often the last line of effective therapy for treating infections caused by multidrug-resistant *Enterobacteriaceae*. Three types of β-lactamases inactivate the carbapenems: *K. pneumoniae* carbapenemases, metallo-β-lactamases (MBLs), and oxacillinases. The 2 most reported MBLs are the VIM and IMP types, which until recently have been mostly associated with *Pseudomonas aeruginosa* and *Acinetobacter* spp., although VIM-2 has spread among *Enterobacteriaceae* in Greece and, to a lesser extent, Italy (*1*).

Recently, a new type of MBL, New Delhi metallo-β-lactamase (NDM-1), in bacteria (*K. pneumoniae* and *E. coli*) recovered from a patient from Sweden who was hospitalized in New Delhi, India, was described (*2*).We characterized a carbapenem-resistant *E. coli* isolate from the urine of a patient with pyelonephritis and prostatitis who returned to Canada after recent hospitalization while visiting India.

## The Study

A 32-year-old man was admitted to the medical ward of a hospital in Mysore, southwestern India, during 2010, with hyperglycemia and upper urinary tract infection (UTI). His underlying diabetes mellitus was stabilized, but his UTI did not improve after 5 days of ciprofloxacin. He was transferred to a hospital in Alberta, Canada. Prostatitis with pyelonephritis was diagnosed, and the patient was treated with ertapenem, 2 g/day. Culture of a clean-catch urine sample taken before the ertapenem was started yielded *E. coli* MH01 at >10^5^ CFU/mL urine. The patient improved clinically, and a urine culture taken after 7 days of therapy showed no bacterial growth. The patient received 1 dose of 3 g fosfomycin after completing the ertapenem.

Antimicrobial drug susceptibility was determined with the VITEK 2 instrument (Vitek AMS; bioMérieux Vitek Systems, Hazelwood, MO, USA). MICs of the following drugs were determined: amoxicillin/clavulanic acid, piperacillin/tazobactam, cefoxitin, ceftriaxone, ceftazidime, aztreonam, meropenem, ertapenem, amikacin, gentamicin, tobramycin, ciprofloxacin, and trimethoprim/sulfamethoxazole. Additional susceptibility tests for imipenem, meropenem, ertapenem, tigecycline, and colistin were performed by using Etest (AB BioDisk, Solna, Sweden) according to the manufacture’s instructions. Results were interpreted by using Clinical and Laboratory Standards Institute (CLSI) criteria for broth dilution (*3*). Fosfomycin susceptibility was determined by using CLSI disk methods (*3*).

The sample with *E. coli* was screened for MBLs with the MBL Etest according to the manufacturer’s instructions. Isoelectric focusing was performed on freeze–thaw extracts on polyacrylamide gels as described (*4*). PCR amplification for *bla*_VIM_, *bla*_IMP_, *bla*_NDM_, *bla*_CTX-Ms_, *bla*_OXAs_, *bla*_TEMs_, and *bla*_SHV_ was conducted on the isolate by using a GeneAmp 9700 ThermoCycler instrument (Applied Biosystems, Norwalk, CT, USA) and PCR conditions and primers as described (*4**–**6*). The *bla*_CTX-M_ was sequenced by using PCR conditions and primers as described (*4*), and the *bla*_NDM_ was sequenced by using the following primers and conditions: NDM-F1: 5′-CAGCGCAGCTTGTCG-3′, NDM-R1: 5′-TCGCGAAGCTGAGCA-3′. The PCR program consisted of an initial denaturation step at 95°C for 5 min; followed by 30 cycles of DNA denaturation at 95°C for 1 min, primer annealing at 52°C for 1 min, and primer extension at 72°C for 1 min; followed by a final extension at 72°C for 5 min.

The *qnrA*, *qnrS*, and *qnrB* genes were amplified in MH01 by using multiplex PCR (*7*). The *aac(6’)-Ib* and *qepA* genes were amplified in a separate PCR by using primers and conditions as described (*8**,**9*). The variant *aac(6′)-Ib-cr* was further identified by digestion with *BstF5*I (New England Biolabs, Ipswich, MA, USA).

Multilocus sequencing typing (MLST) was performed on MH01 by using 7 conserved housekeeping genes (*adk*, *fumC*, *gyrB*, *icd*, *mdh*, *purA*, and *recA*). The MLST protocol, including allelic type and sequence type assignment methods, is detailed at http://mlst.ucc.ie/mlst/dbs/Ecoli.

MH01 was assigned to 1 of the 4 main *E. coli* phylogenetic groups (A, B1, B2, D) by using a multiplex PCR-based method (*10*). Plasmid sizes were determined by using protocols and conditions described (*11*) and assigned to plasmid families by PCR-based replicon typing (*12*). Conjugation experiment was performed by mating-out assays with a selection agar containing different β-lactams (IMP 2 µg/mL, ceftazidime 4 µg/mL respectively) and by using *E. coli* C600N as recipient.

When we used Vitek 2, *E. coli* MH01 was resistant to amoxicillin/clavulanic acid, piperacillin/tazobactam, cefoxitin, ceftriaxone, ceftazidime, aztreonam, meropenem, ertapenem, amikacin, gentamicin, tobramycin, ciprofloxacin, and trimethoprim/sulfamethoxazole. The MICs detected by Etest were meropenem 32 µg/mL, imipenem 32 µg/mL, ertapenem >32 µg/mL, tigecycline 0.5 µg/mL, and colistin 0.125 µg/ml. The zone size for fosfomycin was 26 mm. MH01 was susceptible only to tigecycline and fosfomycin; CLSI has not published colistin MICs for *Enterobacteriaceae*.

*E. coli* MH01 was positive for MBL production by MBL Etest. Isoelectric focusing showed that *E. coli* MH01 produces 2 β-lactamases with isoelectric points of 5.2 and 8.9; PCR with sequencing identified these enzymes as NDM-1 and CTX-M-15, respectively. The isolate was positive for *aac(6′)-Ib* (but not *aac(6′)-Ib-cr*) and belonged to MLST clone 101 and phylogenetic group B1. *E. coli* MH01 harbored 4 plasmids of 75 kb, 165 kb, 300 kb, and 400 kb. *E. coli* (MH01A) transconjugant with an MBL phenotype was obtained, and plasmid analysis showed that it harbored a 75-kb plasmid. PCR confirmed that the transconjugant contained *bla*_NDM_ that was untypeable by PCR-based replicon typing. The *bla*_CTX-M-15_ was identified on the 165-kb plasmid that belonged to incompatibility groups IncA/C and IncFII. These results were similar to those obtained by Poirel et al. (*13*).

## Conclusions

Kumarasamy et al. (*5*) recently provided evidence that NDM-producing *Enterobacteriaceae* (mostly *K. pneumoniae* and *E. coli*) are widespread in the Indian subcontinent. They also found that many patients in the United Kingdom infected with bacteria that produce NDM-1 had been hospitalized on the Indian subcontinent. The patients sought care for a variety of hospital- and community-associated infections; UTIs were the most common clinical infections. NDM-producing *Enterobacteriaceae* also have recently been isolated from patients residing in the United States (*14*), Netherlands (*15*), and Australia (*5*); all patients had received medical care while visiting India.

Our findings add Canada to the growing list of countries from which these bacteria have been isolated. An *E. coli* isolate with NDM-1 and belonging to the same sequence type has been reported from Australia from a patient previously hospitalized in Bangladesh (*13*). Isolation of the same clone in 2 patients in different countries without any obvious contact underscores the probable acquisition of these bacteria during receipt of medical care in the subcontinent and suggests that *E. coli* ST101 with NDM-1 may be widespread throughout the region. The recent pandemic caused by *E. coli* clone ST131, which produces CTX-M types of β-lactamases, highlights the ability of certain clones to spread rapidly. *E. coli* ST101 with NDM-1 may have the potential to cause a similar pandemic.

The worldwide spread of *Enterobacteriaceae*-producing NDMs has serious implications for the empiric treatment of hospital- and community-associated infections because of the multiresistant nature of these bacteria, which severely limits treatment options. Worse, few antimicrobial drugs being developed have activity against gram-negative bacteria. If the emerging public health threat of international travel in the spread of antimicrobial resistance is ignored, the medical community may face carbapenem-resistant *Enterobacteriaceae* that cause common infections such as UTIs.
